# Supplementary material for: Maternal Low-Protein Diet during Puberty and Adulthood Aggravates Lipid Metabolism of Their Offspring Fed a High-Fat Diet in Mice
Source: Nutrients. 2022 Sep 29;14(19):4057. doi: 10.3390/nu14194057 (PMC9570549; doi:10.3390/nu14194057)
Supplement: Supplementary file 1 [file nutrients-14-04057-s001.zip › Supplement figures 0907.pdf]

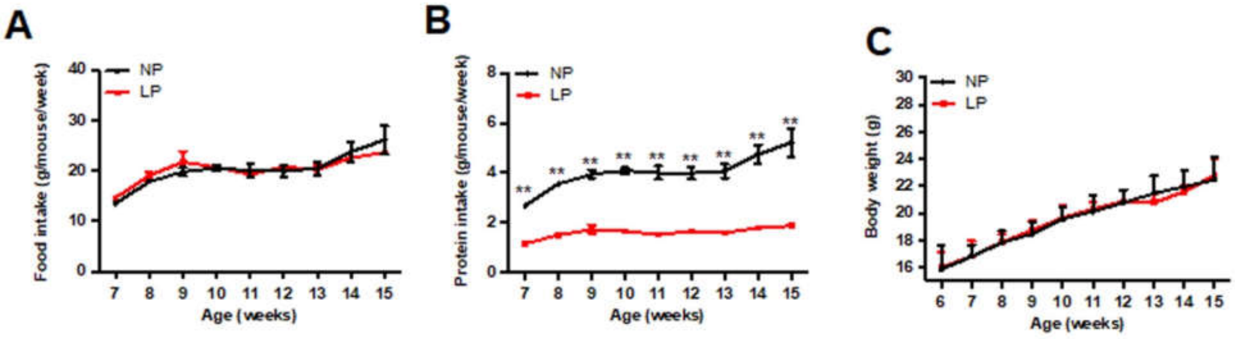

Figure S1. Effect of low protein diet on food intake, protein intake, and body weight in mothers. Female mice were fed with low-protein diet for 11 weeks or normal protein diet. The food intake and body weight were recorded weekly. (A) Food intake. (B) Calculated protein intake. (C) Body weight. (N = 12 for each group). Data were expressed as Mean ± SE. **$P < 0.01$.

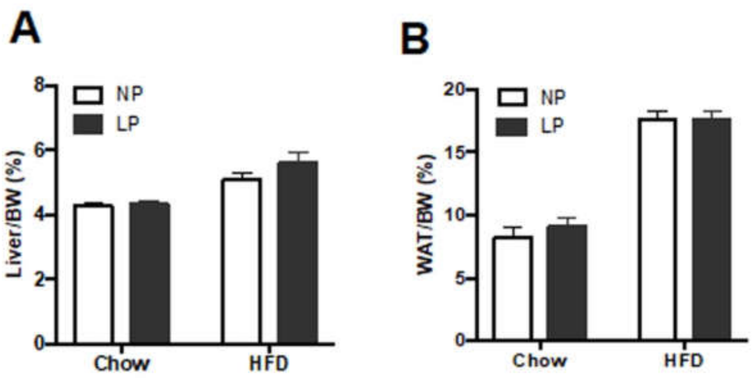

Figure S2. Effect of maternal low-protein diet on liver and white adipose tissue weight in the offspring. (A) Liver weight index. (B) White adipose tissue weight index. N = 7-9 for each group. Data were expressed as Mean ± SE.
